# Comprehensive analysis of the expression, prognostic significance, and regulation pathway of *G2E3* in breast cancer

**DOI:** 10.1186/s12957-022-02871-0

**Published:** 2022-12-15

**Authors:** Yanyan Shen, Jinqi Xue, Jiahui Yu, Yi Jiang, Jiawen Bu, Tong Zhu, Xi Gu, Xudong Zhu

**Affiliations:** 1 Department of Neurosurgery, Shengjing Hospital of China Medical University, Shenyang, 110004 Liaoning China; 2 Department of Oncology, Shengjing Hospital of China Medical University, Shenyang, 110004 Liaoning China; 3 Department of Ultrasound, Shengjing Hospital of China Medical University, Shenyang, 110004 Liaoning China; 4 Department of General Surgery, Cancer Hospital of China Medical University, Liaoning Cancer Hospital and Institute, Shenyang, 110042 Liaoning China

**Keywords:** G2E3, Breast cancer, Prognosis, Therapeutic targets, Tumor immunity

## Abstract

**Background:**

Loss of G2-specific E3-like (G2E3) protein sensitizes tumor cells to chemotherapy. However, the role of G2E3 in breast cancer development and patient’s prognosis is unclear. Here, we explored the expression, prognostic significance, and regulatory pathway of *G2E3* in breast cancer.

**Methods:**

TCGA and UALCAN database were utilized to explore G2E3 expression in breast cancer and normal tissues and its expression in breast cancer based on clinicopathological characteristics, respectively. The Kaplan–Meier plotter database was utilized to determine the effect of G2E3 on the prognosis of breast cancer patients. RT-PCR was utilized to validate the G2E3 expression in cancerous and normal breast tissues. Immunohistochemistry analysis was utilized to validate the prognostic effect of G2E3 expression in breast cancer patients and the relationship between G2E3 expression and lymphocyte infiltration levels. Receiver operating characteristic (ROC) curves were also generated to validate the diagnostic value of G2E3 expression in recurrence/distant organ metastasis and death. The STRING database, DAVID database, and Sanger-box tools were utilized to perform GO functional, KEGG pathway enrichment, and GSEA analysis. The TISIDB database was utilized to determine the relationship between G2E3 expression and tumor immunity. Finally, CTD database was utilized to screen for potential therapeutic compounds that could reduce the *G2E3* mRNA expression.

**Results:**

TCGA data presented that G2E3 expression was higher in breast cancer tissues than in normal breast tissues. This result was further validated by RT-PCR (*P* = 0.003). The Kaplan–Meier plotter database suggested that patients with high G2E3 mRNA expression had significantly shorter RFS and OS than patients with low G2E3 mRNA expression. Immunohistochemistry analysis of 156 breast cancer clinical specimens also validated patients with G2E3-positive expression had a significantly shorter DFS and OS than patients with G2E3-negative expression. Thus, G2E3 expression was an independent prognostic predictor of DFS and OS. The G2E3-positive expression also has a high diagnostic value for recurrence/distant organ metastasis and death. GSEA analysis revealed that G2E3 might be enriched in the E2F, PI3K/AKT/mTOR signaling, DNA repair pathways, and other cancer-related signaling pathways. The TISIDB database showed that G2E3 expression was significantly negatively associated with lymphocyte infiltration. This result was further validated in clinical breast cancer samples (*P* = 0.048; *R* = −0.158). Using the CTD database, we found that (+)-JQ1 compound, 1,2-dimethylhydrazine, and other compounds may decrease the G2E3 mRNA expression. These compounds could serve as potential therapeutic compounds for the clinical treatment of breast cancer.

**Conclusions:**

G2E3 expression was higher in breast cancer tissues than in normal tissues. G2E3-positive expression was related to a worse survival outcome in patients with breast cancer. Genes co-expressed with G2E3 may be enriched in the breast cancer-related signaling pathways. The G2E3 expression was significantly negatively associated with lymphocyte infiltration. G2E3 may serve as a novel prognostic biomarker and therapeutic target for breast cancer.

**Supplementary Information:**

The online version contains supplementary material available at 10.1186/s12957-022-02871-0.

## Introduction

Breast cancer has been a malignant tumor with the highest morbidity in women. New breast cancer cases account for approximately 30% of the total number of new malignant tumors in women every year, which is a significant threat to their health [1]. Although breast cancer molecular typing and the emergence of comprehensive treatment methods have greatly improved the patient survival outcomes [2, 3], local recurrence and distant metastasis remain the leading causes of breast cancer-related death [4, 5]. Breast cancer was initially considered a local disease. However, they can migrate into lymph nodes and distant target organs, such as the bone, lung, and liver [6–9]. Current adjuvant therapy for breast cancer can also eliminate tumor cells that have spread to distant sites at the time of diagnosis and significantly improve the 10-year survival rate of women. However, about 40% of women who have already received adjuvant therapy would develop postoperative metastasis and eventually die of metastatic breast cancer [5, 10]. Therefore, identifying the intrinsic driver genes of tumor metastasis, screening effective therapeutic targets, and developing targeted drugs may prevent tumor metastasis from the root cause [7, 11].

G2-specific E3-like (G2E3) protein was first identified by Brooks et al. [12]. It is denoted as G2E3 as it is a G2-specific protein with a domain similar to many E3 proteins. Schmidt et al. found that G2E3 may be a potential target for chemo-sensitizing tumor cells and might participate in response to cisplatin [13]. G2E3 knockdown may promote apoptosis and inhibit cancer cell proliferation. G2E3 can be a molecular determinant of the DNA damage response (DDR) and cell survival, and its loss of expression can sensitize tumor cells to chemotherapy. However, the role of G2E3 in breast cancer development and patient’s prognosis is still unclear. Therefore, we explored the prognostic significance of G2E3 expression in breast cancer patients and the effect of G2E3 on the malignant biological behavior of cancer cells.

This study primarily analyzed the G2E3 expression in cancerous and normal breast tissues, explored the effect of G2E3 high expression on patient’ survival outcomes, and validated it using clinical breast cancer samples. Furthermore, we explored genes co-expressed with *G2E3* and performed GO functional and KEGG pathway enrichment analyses. Following this, we analyzed the relationships between G2E3 expression, tumor immunity, and mutations of *G2E3* in breast cancer. Finally, potential therapeutic compounds that can decrease the *G2E3* mRNA expression in breast cancer were screened. Therefore, we identified an effective therapeutic target for breast cancer.

## Methods and materials

### GTEx database

The Genotype-Tissue Expression (GTEx) database (www.gtexportal.org) was utilized to determine *G2E3* expression in 31 normal human tissues [14].

### CCLE database

CCLE database (https://sites.broadinstitute.org/ccle) was utilized to determine the *G2E3* expression in a series of cancer cell lines [15].

### UALCAN database

UALCAN database (ualcan.path.uab.edu/index.html) was utilized to explore the *G2E3* expression in breast cancer patients based on sample type, patient age, individual cancer stage, menopause status, nodal metastasis status, and breast cancer subclasses [16].

### Kaplan–Meier plotter database

The Kaplan–Meier plotter database (http://kmplot.com) was utilized to determine the effect of high *G2E3* mRNA expression on relapse-free survival (RFS) and overall survival (OS) in breast cancer patients [17].

### RT-qPCR

Total RNA from 30 pairs of fresh cancerous and normal breast tissues were isolated using TRIzol solution (Solarbio Company). The extracted RNA was reverse-transcribed by a cDNA synthesis kit (TaKaRa). qPCR was performed by SYBR Green PCR Master Mix (TaKaRa) and primers binding to the *G2E3* and *GAPDH*. These primers were designed by Shanghai Sangon Biotech Co., Ltd. (*G2E3*: forward, 5′-CAGCACTATGAGCGTTGTGATGTTC-3′ and reverse 5′-ACCGTAATGAGGAGCAGGCTAAATG-3′; *GAPDH*: forward, 5′-CCTTCCGTGTCCCCACT-3′ and reverse, 5′-GCCTGCTTCACCACCTTC-3′). The cycling protocol was 95 °C for 30 s (initial denaturation), followed by 40 denaturation cycles at 95 °C for 3 s, and finally annealing and extension at 60 °C for 30 s. Relative mRNA levels were calculated using the 2^−ΔΔCt^ method [18]. These breast cancer specimens were obtained from affiliated hospital of China Medical University at the time of surgery, and the basic clinicopathological characteristics of the 30 patients were presented in Supplementary Table 1. This study was approved by China Medical University Institutional Review Board.

### Patients and breast cancer specimens

This study also included 156 patients diagnosed with invasive ductal carcinoma between January 2007 and November 2011 at affiliated hospital of China Medical University. All patients underwent routine operation and postoperative treatment. The inclusion criteria were as follows: (1) complete clinicopathological patient information was required and (2) no organ metastasis at the time of surgery. The exclusion criteria were as follows: (1) patients who did not have complete clinicopathological information, (2) patients who did not receive surgery or routine postoperative treatment, and (3) patients with unknown survival status. All patients were followed up for at least 10 years. Disease-free survival (DFS) was defined from the date of the operation to the date of recurrence/ metastases. OS was defined as the time from operation to death. The survival status of patients was determined using interviews/telephone calls.

### Immunohistochemistry analysis

The clinical specimens obtained from 156 patients were fixed and embedded in paraffin. Then, they were sliced into 4-μm sections and were deparaffinized and rehydrated. The sections were incubated with an antibody against G2E3 (1:200; Bioss, bs-17000R) at 4 °C overnight. Then, these sections were incubated with a secondary antibody (Zhong Shan Jin Qiao) on the 2nd day at room temperature.

G2E3 expression was semiquantitatively scored as follows: 0, if < 1% of cancer cells expressed G2E3; 1+, if G2E3 was expressed in ≥ 1% to < 5% of cancer cells; 2+ if G2E3 was expressed in ≥ 5 to < 10% of cancer cells; and 3+ if G2E3 was expressed in ≥ 10% of breast cancer cells. Scores of 1+, 2+, and 3+ were considered G2E3 positive.

Evaluation of the levels of tumor-infiltrating lymphocytes was performed as previously described [19].

### STRING analysis and GO functional and KEGG pathway enrichment

The STRING database (https://string-db.org/) was applied to identify genes co-expressed with *G2E3* [20]. Protein-protein interactions (PPI) were also determined. The DAVID database (https://david.ncifcrf.gov) was utilized for GO functional and KEGG pathway enrichment analyses of genes co-expressed with *G2E3* [21].

### TISIDB analysis

The TISIDB database (http://cis.hku.hk/TISIDB) was applied to explore the relationships between G2E3 and tumor immunity [22].

### Sanger-box tools

Sanger-box tools (http://www.sangerbox.com/tool) were used for the following analysis: Gene Set Enrichment Analysis (GSEA); the relationships between G2E3 expression and ImmuneScore, StromalScore, and ImmuneScore; the relationships between G2E3 expression and immune checkpoint gene expression; the relationships between G2E3 expression and the number of tumor neoantigens, tumor mutational burden (TMB), microsatellite instability (MSI), and the mutation pattern of *G2E3* in breast cancer; and the correlations between the expression of *G2E3* in each tumor sample and that of the DNA repair genes (MMRs) and methyltransferase.

### Comparative Toxicogenomics Database (CTD)

CTD is an innovative online database that provides literature-based data on the interactions between oncogenes and chemotherapeutic compounds. This tool was used to screen potential therapeutic compounds that could reduce *G2E3* mRNA expression [23].

### Statistical analysis

Correlations between G2E3 expression and age, tumor (T) grade, lymph nodes metastases (N) grade, histological grade, subtypes, menopausal status, relapse/metastasis, and death were analyzed using the chi-square test. Cox regression analyses were performed to identify independent predictors of DFS and OS. Hazard ratios (HRs) and 95% confidence intervals (CIs) were also calculated. The correlations between G2E3 expression and tumor-infiltrating lymphocytes (TILs) were analyzed using Pearson correlation. Survival curves were generated using the Kaplan-Meier test in SPSS 25.0. ROC curves were generated using SPSS 25.0 software. The significance was set at *P* < 0.05.

## Results

### G2E3 expression in pan-cancer

**Fig. 1:**
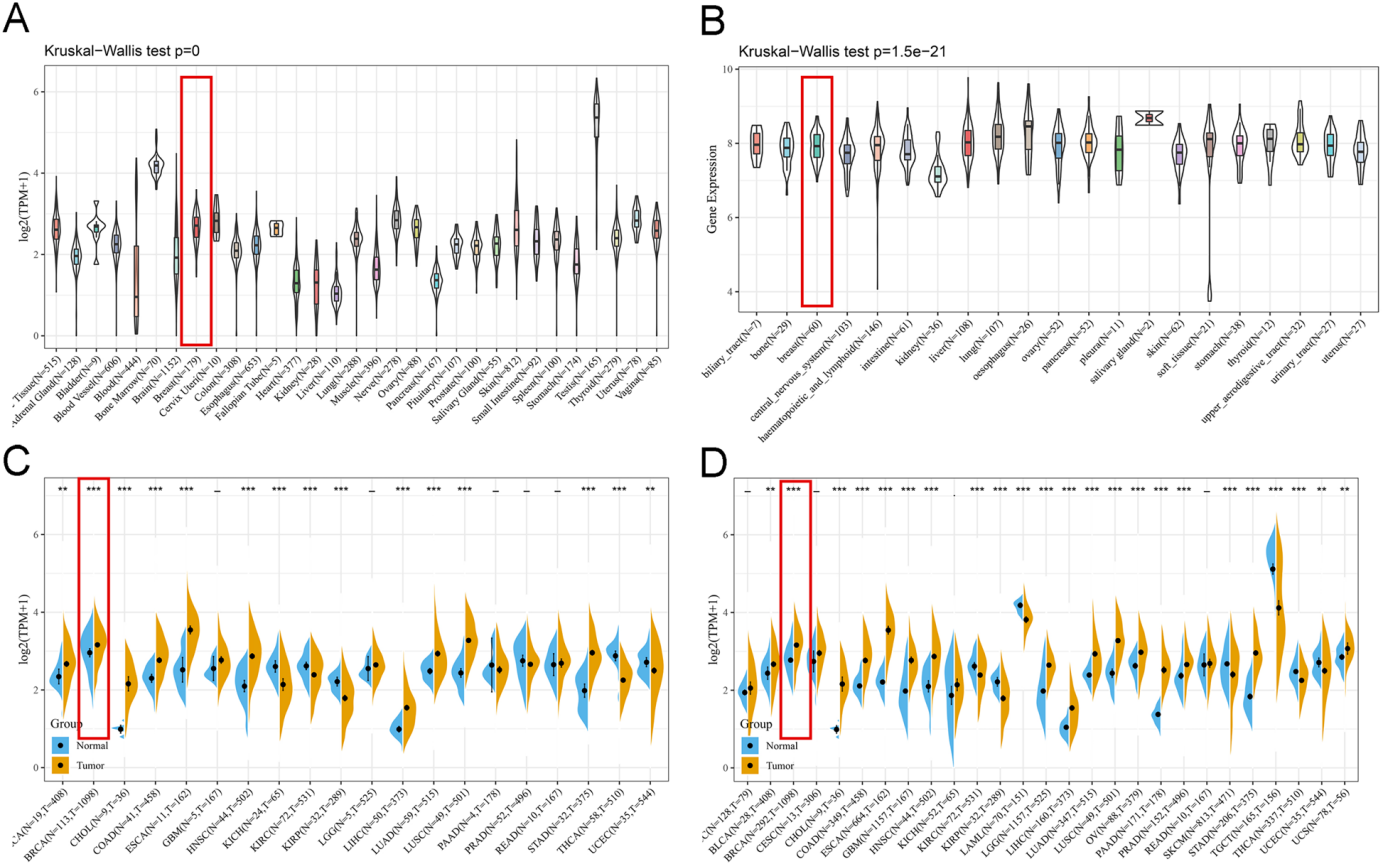
*G2E3* gene expression in pan-cancer. **A** Expression of *G2E3* in 31 normal human tissues using the GTEx database. **B** Expression of *G2E3* in 21 tissue types. **C ***G2E3* expression between cancer tissues and adjacent normal tissues by TCGA database. **D ***G2E3* expression between cancer tissues and adjacent normal tissues in each tumor sample integrated with the data from the GTEx and TCGA databases

Figure 1A presents the *G2E3* expression in 31 normal human tissues using the GTEx database. We analyzed the data for a series of cell lines obtained from CCLE database. According to the organization source, the data could be classified into 21 organizations. We analyzed *G2E3* expression in these 21 tissues (Fig. 1B). We then obtained the difference in *G2E3* expression between cancer tissues and normal tissues in each tumor sample from The Cancer Genome Atlas (TCGA) database (Fig. 1C). Because there were fewer normal breast samples in TCGA, we further integrated the data of normal breast tissues from the GTEx database and the data of TCGA tumor tissues to analyze the *G2E3* expression differences among the 27 tumor types (Fig. 1D). Our interest lies in breast cancer research. From the analysis depicted in Fig. 1 A and B, we found that the *G2E3* gene has moderate expression in breast tissues compared with other human tissues. The expression of G2E3 was significantly higher in breast cancer tissues than in normal tissues, shown in Fig. 1 C and D.

### Expression and prognostic effect of G2E3 in breast cancer explored by UALCAN and Kaplan–Meier plotter databases

**Fig. 2:**
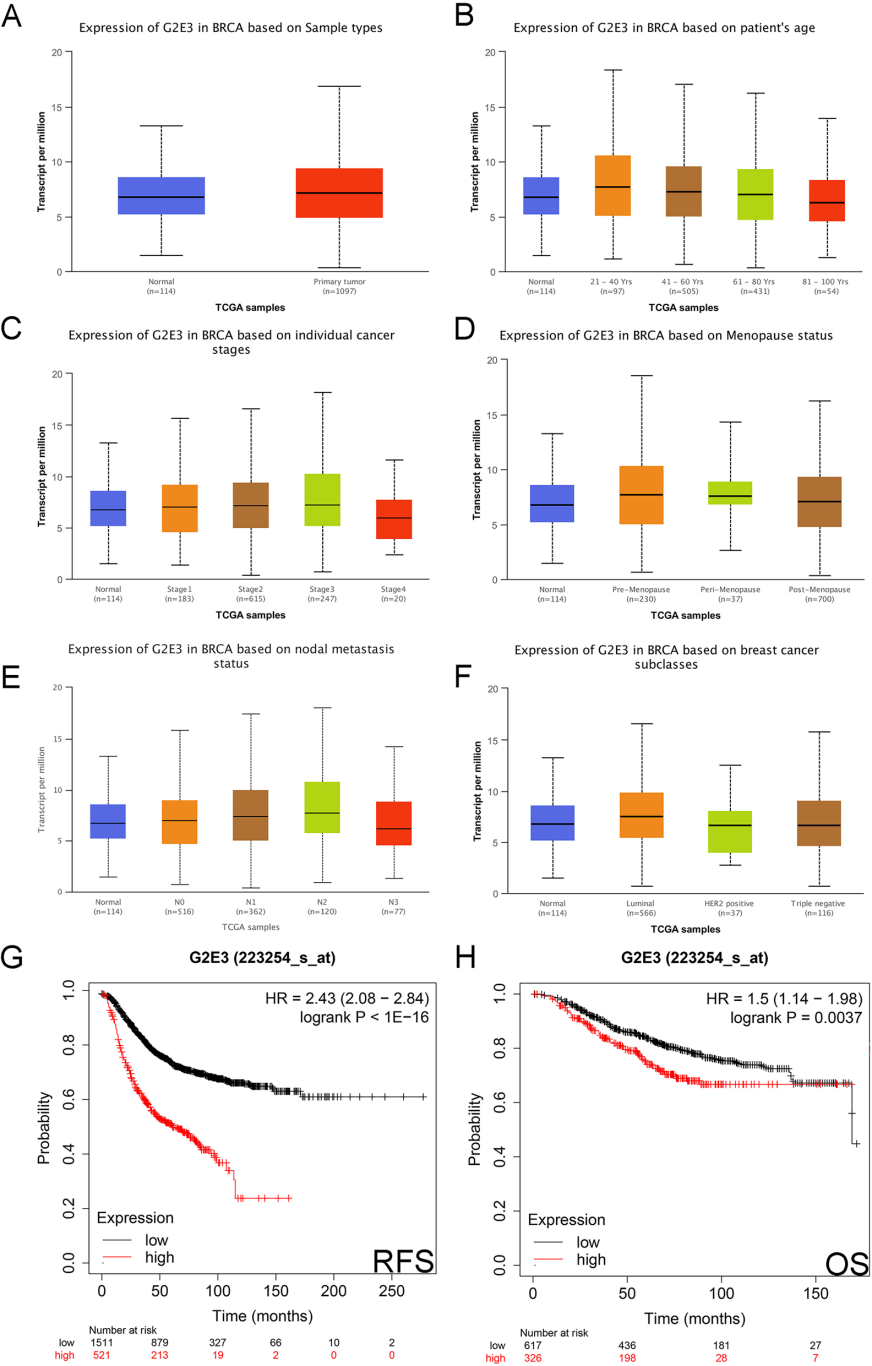
Expression and prognostic effect of G2E3 in breast cancer explored by UALCAN and Kaplan–Meier plotter database. **A** The expression of G2E3 based on sample type. **B** The expression of G2E3 based on the patient’s age. **C** The expression of G2E3 based on individual cancer stages. **D** The expression of G2E3 based on menopause status. **E** The expression of G2E3 based on nodal metastasis status. **F** The expression of G2E3 based on breast cancer subclasses. **G** The effect of G2E3 expression on RFS in patients with breast cancer. **H** Effect of G2E3 expression on the OS of patients with breast cancer

Using the UALCAN database, we also found the expression of *G2E3* mRNA was significantly higher in breast cancer than in normal tissues (*P* = 1.96E−03; Fig. 2A). In the subgroup of patients, those aged 21–40 years had the highest *G2E3* mRNA expression (*P* = 4.68E−03; Fig. 2B). In the subgroup of individual cancer stages, patients in stage 3 had the highest *G2E3* mRNA expression (*P* = 1.82E−03; Fig. 2C). In the subgroup of patients with menopausal status, those who were premenopausal had the highest *G2E3* mRNA expression (*P* = 5.79E−04; Fig. 2D). In the subgroup of patients with nodal metastasis, N2 patients had the highest *G2E3* mRNA expression (*P* = 1.60E−04; Fig. 2E). Finally, in the subgroup of patient subclasses, patients with luminal cancer had the highest *G2E3* mRNA expression (*P* = 1.24E−03; Fig. 2F).

Using the Kaplan–Meier plotter, we preliminarily found high *G2E3* expression had a significant adverse effect on RFS (*HR* = 2.43 (2.08–2.84); *P* < 1E−16; Fig. 2G) and OS (*HR* = 1.5 (1.14–1.98); *P* = 0.0037; Fig. 2H).

### Validation of the expression and prognostic effect of G2E3 in breast cancer clinical specimens

**Fig. 3:**
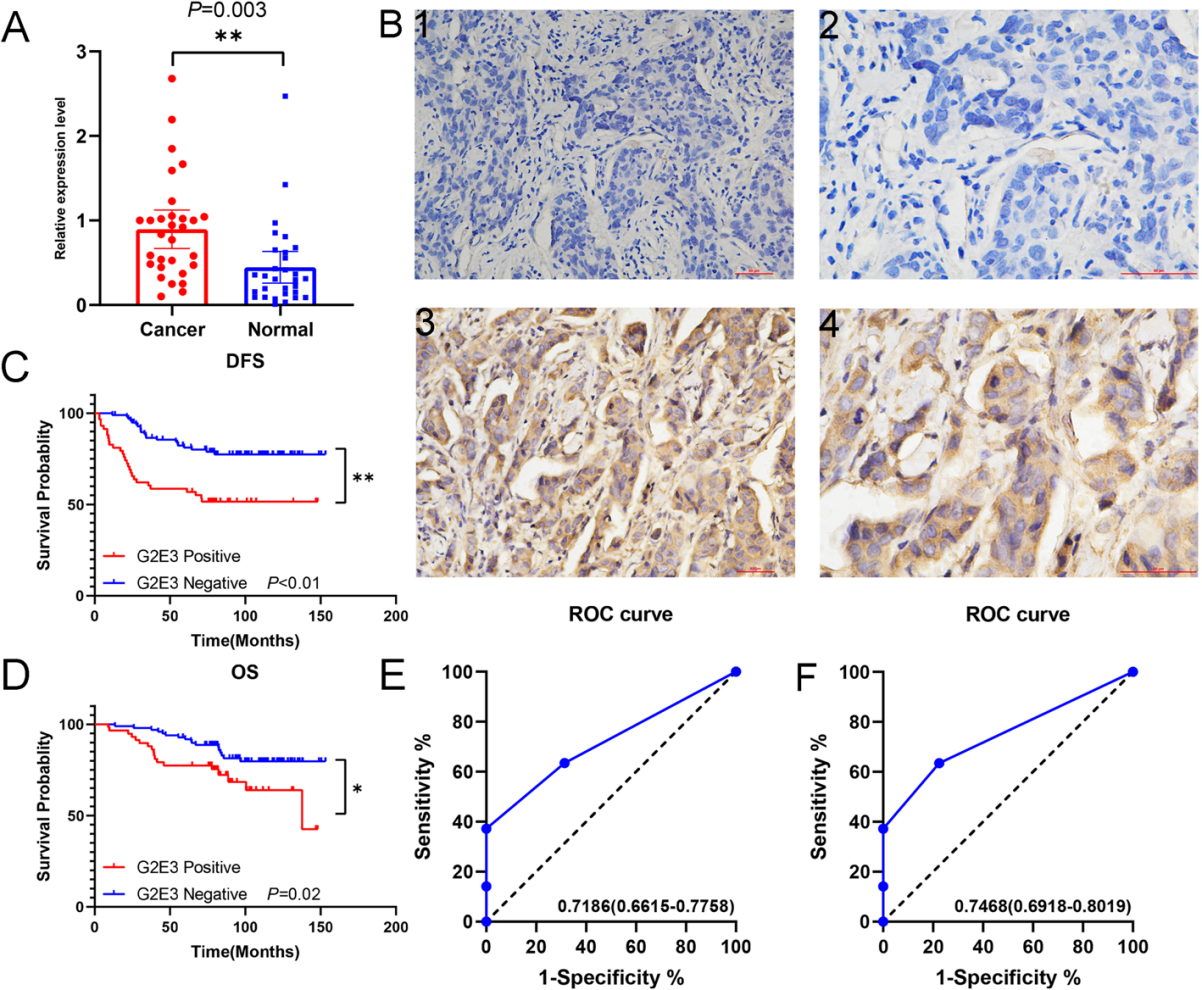
Validation of the expression and prognostic effect of G2E3 in breast cancer by breast cancer clinical specimens. **A** Validating the expression of G2E3 in breast cancer and normal breast tissues by RT-PCR. **B** Representative images of immunohistochemical analysis of G2E3 in clinical breast cancer specimens. 1, negative expression of G2E3 (200×); 2, negative expression of G2E3 (400×); 3, positive expression of G2E3 (200×); 4, positive expression of G2E3 (400×). **C** The effect of G2E3 expression on DFS in 156 patients with breast cancer. **D** The effect of G2E3 expression on OS in 156 patients with breast cancer. **E** The diagnostic value of G2E3 expression for recurrence/distant organ metastasis. **F** The diagnostic value of G2E3 expression for death

First, we validated the expression of *G2E3* mRNA in 30 pairs breast cancer and normal tissues by RT-PCR. We found *G2E3* mRNA expression was significantly higher in breast cancer tissues than in normal tissues (*P* = 0.003; Fig. 3A). We explored the G2E3 expression by immunohistochemistry in 156 breast cancer specimens, presenting as G2E3-positive and -negative cells (Fig. 3B). We found that patients with G2E3-positive expression had a significantly shorter DFS (*P* < 0.01; Fig. 3C) and OS (*P* = 0.02; Fig. 3D) than patients with G2E3-negative expression. Therefore, we can conclude that G2E3-positive expression has adverse prognostic effects on the prognosis of breast cancer patients. We also explored the diagnostic value of G2E3 expression in recurrence/distant organ metastasis and death. For recurrence/distant organ metastasis, G2E3 expression had a high diagnostic value, wherein the area under the curve (AUC) was 0.7186 (*P* < 0.001; Fig. 3E). G2E3 expression also had a high diagnostic value for death, with an AUC of 0.7468 (*P* < 0.001; Fig. 3F).

We further analyzed the relationships between G2E3 expression and age, T grade, N grade, histological grade, subtypes, menopausal status, relapse/metastasis, and death in these 156 patients with breast cancer. We observed that G2E3-positive expression was related to a higher N grade, although the difference was not significant (*P* = 0.055). Moreover, G2E3 expression was significantly related to the occurrence of relapse/distant organ metastasis (*P* < 0.001) and death (*P* = 0.048). No other positive results were observed. The results are displayed in Table 1.

**Table 1:** The correlations between G2E3 expression and clinicopathological characteristics

Variables	G2E3 positive (%)	G2E3 negative (%)	*p*-value
No. of patients	58 (37.2)	98 (62.8)	
Age			0.185
≤ 45	49 (84.5)	74 (75.5)	
> 45	9 (15.5)	24 (24.5)	
T grade			0.537
1	13 (22.4)	19 (19.4)	
2	44 (75.9)	74 (75.5)	
3	1 (1.7)	5 (5.1)	
N grade			0.055
0	25 (43.1)	59 (60.2)	
1	15 (25.9)	23 (23.5)	
2	6 (10.3)	9 (9.2)	
3	12 (20.7)	7 (7.1)	
Histological grade			0.885
1	2 (3.4)	5 (5.1)	
2	42 (72.4)	69 (70.4)	
3	14 (24.1)	24 (24.5)	
Subtypes			0.050
Luminal	39 (67.2)	52 (53.1)	
HER2 positive	13 (22.4)	20 (20.4)	
Triple negative	6 (10.4)	26 (26.5)	
Menopausal status			0.668
Premenopausal	24 (41.4)	44 (44.9)	
Postmenopausal	34 (58.6)	54 (55.1)	
Relapse/distant organ metastasis			< 0.001
Yes	28 (48.3)	21 (21.4)	
No	30 (51.7)	77 (78.6)	
Death			0.048
Yes	18 (31.0)	17 (17.3)	
No	40 (69.0)	81 (82.7)	

To identify independent predictors of prognosis, we performed Cox regression analysis. These results were displayed in Tables 2 and 3.

**Table 2:** Univariate and multivariate Cox regression analyses of clinicopathological factors for DFS among these breast cancer patients

Variables	DFS			
	Univariate analysis		Multivariate analysis	
	HR (95% *CI*)	*p*-value	HR (95% *CI*)	*p*-value
Age	1.478 (0.693–3.152)	0.313	NA	
T grade			NA	
1		0.644		
2	1.434 (0.670–3.069)	0.353		
3	1.492 (0.317–7.027)	0.613		
N grade				
0		< 0.001		< 0.001
1	3.392 (1.567–7.342)	0.002	3.241 (1.496–7.021)	0.003
2	5.481 (2.302–13.048)	< 0.001	5.613 (2.352–13.395)	< 0.001
3	8.928 (4.081–19.533)	< 0.001	6.899 (3.102–15.346)	< 0.001
Histological grade			NA	
1		0.821		
2	1.189 (0.285–4.957)	0.812		
3	1.415 (0.322–6.230)	0.646		
Menopausal status	1.073 (0.609–1.890)	0.806	NA	
G2E3 expression	2.954 (1.676–5.207)	< 0.001	2.395 (1.332–4.306)	0.004

*NA* non-analysis

**Table 3:** Univariate and multivariate Cox regression analyses of clinicopathological factors for OS among these breast cancer patients

Variables	OS			
	Univariate analysis		Multivariate analysis	
	HR (95% *CI*)	*p*-value	HR (95% *CI*)	*p*-value
Age	1.389 (0.576–3.345)	0.464	NA	
T grade			NA	
1		0.698		
2	1.499 (0.580–3.879)	0.403		
3	1.216 (0.142–10.409)	0.859		
N grade				
0		0.001		0.003
1	2.473 (1.029–5.942)	0.043	2.310 (0.957–5.574)	0.062
2	3.130 (1.062–9.225)	0.039	3.040 (1.030–8.977)	0.044
3	6.175 (2.562–14.883)	< 0.001	5.319 (2.168–13.049)	< 0.001
Histological grade			NA	
1		0.251		
2	1.775 (0.236–13.358)	0.577		
3	2.992 (0.389–23.019)	0.293		
Menopausal status	1.507 (0.758–3.000)	0.242	NA	
G2E3 expression	2.223 (1.143–4.325)	0.019	1.780 (0.902–3.512)	0.096

*NA* non-analysis

For DFS, N grade (*P* < 0.001) and G2E3 expression (*P* = 0.004) were considered independent predictors of DFS in these 156 patients with breast cancer. For OS, the N grade was an independent predictor of OS (*P* = 0.003). However, G2E3 expression was not a significant independent predictor of overall with OS (*P* = 0.096). These results suggest that G2E3 is an independent predictor of DFS, but not OS.

### PPI network construction and GO functional and KEGG pathway enrichment analysis

**Fig. 4:**
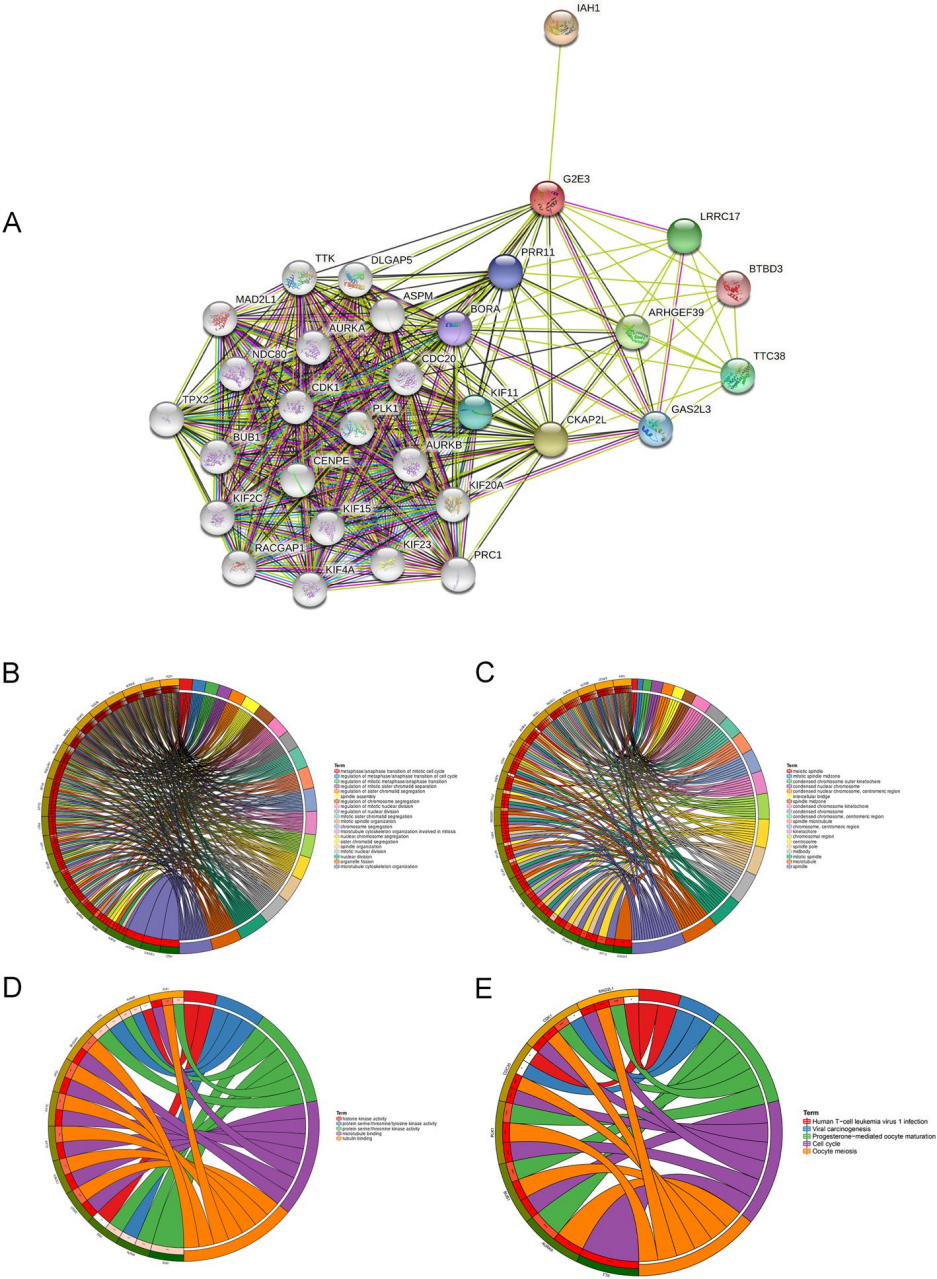
PPI network construction and GO functional and KEGG pathway enrichment analysis of genes co-expressed with G2E3. **A** PPI network of genes co-expressed with G2E3. **B**–**E** GO functional and KEGG pathway enrichment analyses based on the co-expressed genes using the DAVID database. **B** BP; **C** CC; **D** MF; **E** KEGG pathway enrichment analysis

Using the STRING database, we constructed a PPI network of co-expressed genes with G2E3 (Fig. 4A). These genes are *IAH1*, *LRRC17*, *ARHGEF39*, and *PRR11*. We then performed functional and pathway enrichment analyses based on these genes using the DAVID database. GO functional enrichment analysis consisted of biological process (BP), cellular component (CC), and molecular function (MF). For BP, these genes were mainly enriched in processes such as mitotic cells’ metaphase/anaphase transition and regulation of the metaphase/anaphase transition of the cell cycle (Fig. 4B). For CC, these genes were mainly enriched in components such as meiotic spindles, condensed chromosome outer kinetochores, and intercellular bridges (Fig. 4C). For MF, these genes were mainly enriched in histone kinase activity and protein serine/threonine/tyrosine kinase activity (Fig. 4D). KEGG pathway analysis suggested that they were mainly enriched in cell cycle and oocyte meiosis (Fig. 4E).

To further explore the potential regulation pathways of G2E3 expression in breast cancer, GSEA analysis was performed between the G2E3 high- and low-expression groups. The top 10 phenotypes are presented in Supplementary Fig. 1 and Table 4. They were “HALLMARK_G2M_CHECKPOINT,” “HALLMARK_E2F_TARGETS,” “HALLMARK_MITOTIC_SPINDLE,” “HALLMARK_MTORC1_SIGNALING,” “HALLMARK_MYC_TARGETS_V1,” “HALLMARK_UNFOLDED_PROTEIN_RESPONSE,” “HALLMARK_PROTEIN_SECRETION,” and “HALLMARK_PI3K_AKT_MTOR_SIGNALING.”

**Table 4:** Gene sets enriched in phenotype high

MSigDB collection	Gene set name	NES	NOM *p*-value	FDR *q*-value
h.all.v6.2.symbols.gmt [Hallmarks]	HALLMARK_G2M_CHECKPOINT	−2.23	0.000	0.002
	HALLMARK_E2F_TARGETS	−2.19	0.000	0.002
	HALLMARK_MITOTIC_SPINDLE	−2.15	0.000	0.003
	HALLMARK_MTORC1_SIGNALING	−2.13	0.000	0.003
	HALLMARK_MYC_TARGETS_V1	−2.10	0.000	0.005
	HALLMARK_UNFOLDED_PROTEIN_RESPONSE	−2.01	0.002	0.011
	HALLMARK_PROTEIN_SECRETION	−2.00	0.000	0.011
	HALLMARK_PI3K_AKT_MTOR_SIGNALING	−1.95	0.000	0.015
	HALLMARK_DNA_REPAIR	−1.92	0.008	0.016
	HALLMARK_SPERMATOGENESIS	−1.92	0.000	0.015

Gene sets with NOM *P*-value < 0.05 and FDR *q*-value < 0.25 were considered as significant

*NES* normalized enrichment score, *NOM* nominal, *FDR* false discovery rate

### Relationships between G2E3 and tumor immunity in breast cancer

**Fig. 5:**
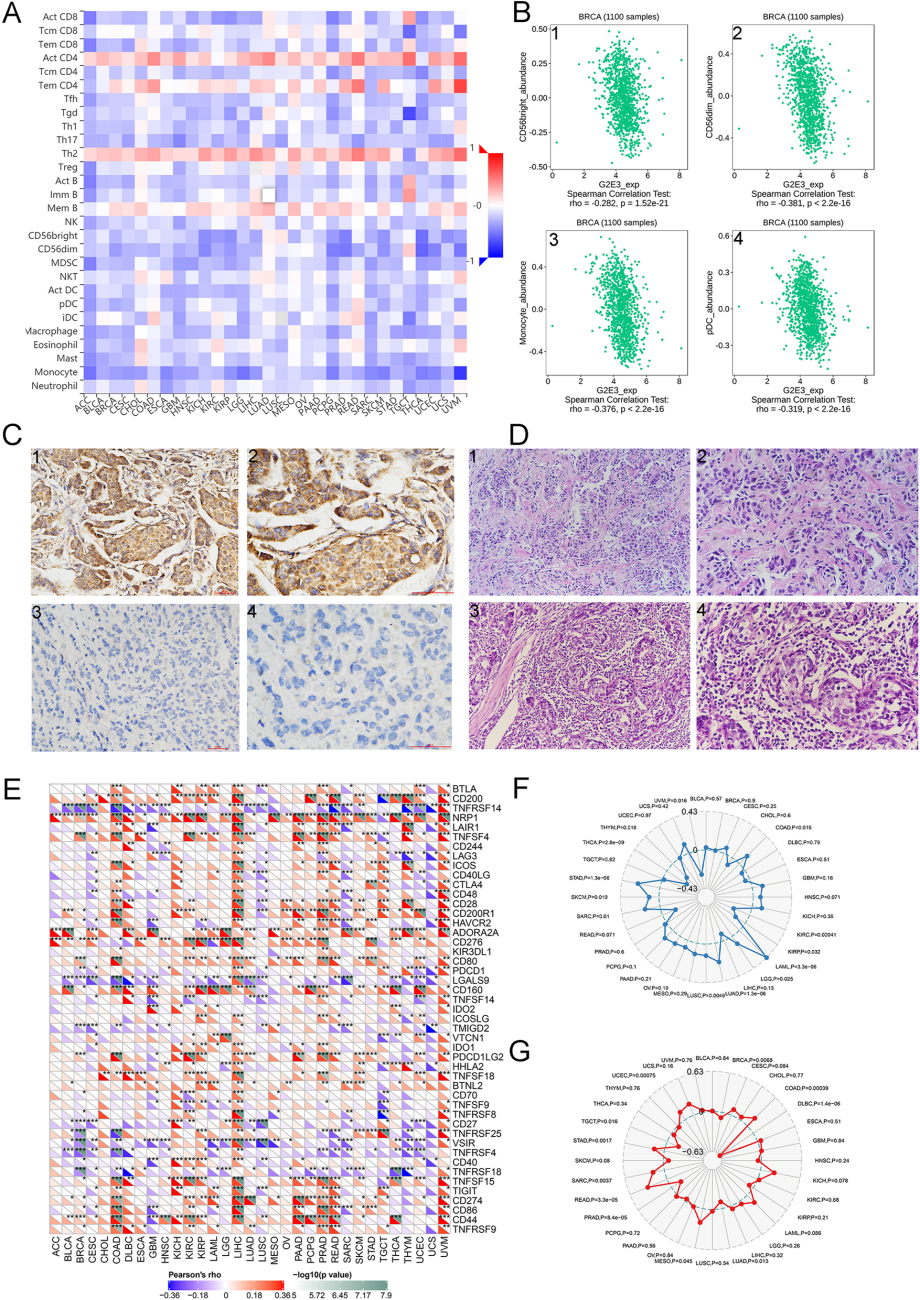
Relationships between G2E3 and immunity in breast cancer. **A** Relationships between G2E3 expression and tumor infiltrative lymphocytes. **B** Top 4 tumor-infiltrating lymphocytes showing the highest correlations with G2E3 expression. **C** Representative images of immunohistochemical analysis of G2E3 in clinical breast cancer specimens. 1, positive expression of G2E3 (200×); 2, positive expression of G2E3 (400×); 3, negative expression of G2E3 (200×); 4, negative expression of G2E3 (400×). **D** Representative images of immunohistochemical analysis of tumor-infiltrating lymphocytes in clinical breast cancer specimens. 1, low levels of tumor infiltrative lymphocytes (200×); 2, low level of tumor-infiltrating lymphocytes (400×); 3, high levels of tumor-infiltrating lymphocytes (200×); 4, high levels of tumor-infiltrating lymphocytes (400×). **E** The relationship between G2E3 expression and immune checkpoint gene expression. **F**–**G** The relationships between *G2E3* gene expression and TMB and MSI in breast cancer

Tumor immunity has been found to be involved in the development of breast cancer [24–26]. Therefore, we evaluated the correlations between G2E3 and immunity in breast cancer. Using the TISIDB database, we first explored the correlation between G2E3 expression and lymphocyte infiltration (Fig. 5A). The top four lymphocytes negatively correlated to G2E3 expression most significantly were CD56 bright, CD56dim, monocyte, and pDC (Fig. 5B). We further experimentally validated the relationship between G2E3 expression and TILs levels in breast cancer clinical samples. We found G2E3 expression was significantly negatively associated with the TILs levels (*P* = 0.048; *R* = −0.158; Table 5). Typical images of G2E3 expression and TILs were shown in Fig. 5 C and D.

**Table 5:** The relationships between G2E3 expression and the level of tumor-infiltration lymphocytes (TILs)

	G2E3 expression			*p*-value	*R*-value
The level of TILs	Negative	Positive	Total		
Low	63	46	109	0.048	−0.158
High	35	12	47		
Total	98	58	156		

We also explored the correlations between G2E3 expression and immunomodulators and chemokines by the TISIDB database. Supplementary Fig. 2A shows the correlation between G2E3 expression and immune-inhibitors. The top four immune-inhibitors most significantly negatively associated with G2E3 expression were LGALS9, PDCD1, TGFβ1, and TGFBR1 (Supplementary Fig. 2B). Supplementary Fig. 2C shows the correlations between G2E3 expression and immune-stimulators. The top four immune-stimulators most significantly negatively associated with G2E3 expression were C10orf54, TNFRSF14, TNFRSF25, and TNFSF4 (Supplementary Fig. 2D). Supplementary Fig. 2E shows relationships between G2E3 expression and major histocompatibility complex (MHC) molecules. The top four MHC molecules most significantly negatively related to G2E3 expression were human leukocyte antigen (HLA)-A, HLA-DPB1, HLA-F, and TAP-binding protein (TAPBP) (Supplementary Fig. 2F). Supplementary Fig. 3A shows the correlation between G2E3 expression and chemokines. The top four chemokines most significantly negatively related to G2E3 expression were CCL14, CCL19, CCL21, and CX3CL1 (Supplementary Fig. 3B). Supplementary Fig. 3C showed the correlation between G2E3 expression and chemokine receptors. The top four chemokine receptors most significantly negatively associated with G2E3 expression were C-C motif chemokine receptor (CCR)7, CCR10, CXCR3, and CXCR5 (Supplementary Fig. 3D). We also explored the expression of G2E3 in different molecular and immune subtypes. The luminal B subtype had the highest G2E3 expression (*P* = 9.33E−17; Supplementary Fig. 3E), similar to the results obtained from the UALCAN database (Fig. 2F). The expression of G2E3 was similar in all immune subtypes of breast cancer (Supplementary Fig. 3F).

An increasing number of reports have indicated that the tumor immune microenvironment plays a vital role in the development of cancer [27–29]. Sanger-box tools provided the analysis of the immune and stromal scores of every tumor sample to observe the correlations between gene expression and immune scores. These data included ImmuneScore, correlations between gene expression and matrix score such as StromalScore, and the correlations between gene expression and ESTIMATE immune score such as ESTIMATE score in 33 tumors. We found that in breast cancer, G2E3 expression was significantly negatively associated with ImmuneScore (Supp. Fig. 4) and ESTIMATE score (Supplementary Fig. 6) but was not significantly related to StromalScore (Supplementary Fig. 5).

Under normal conditions, the immune system can effectively recognize and eliminate tumor cells [30]. However, tumor cells can adopt many strategies to suppress the body’s immune system and prevent tumor cells from being eliminated [31]. All stages of the immune response survived. Tumor immunotherapy is a treatment method that can restore a normal immune response in the body [32, 33]. Figure 5E depicts the analysis from Sangerbox tools that collected nearly 40 immune checkpoint genes and explored the correlations between G2E3 and immune checkpoint gene expression, such as TNFRSF14, NRP1, and CD44.

Tumor neoantigens are abnormal proteins encoded by genetic point and deletion mutations in cancer cells [34, 35]. Using the immune activity of tumor neoantigens, neoantigen vaccines can be designed and synthesized according to mutations in tumor cells, and patients can be immunized to achieve therapeutic effects. Here, Sanger-box tools count the number of neoantigens in every tumor sample and analyze the correlation between G2E3 gene expression and the number of antigens. Unfortunately, there was no significant relationships between G2E3 expression and the number of breast cancer neoantigens (Supplementary Fig. 7). Sanger-box tools also revealed the relationship between G2E3 gene expression and TMB and MSI in breast cancer. These results are shown in Fig. 5F and G.

### Mutations of G2E3 gene in breast cancer

**Fig. 6:**
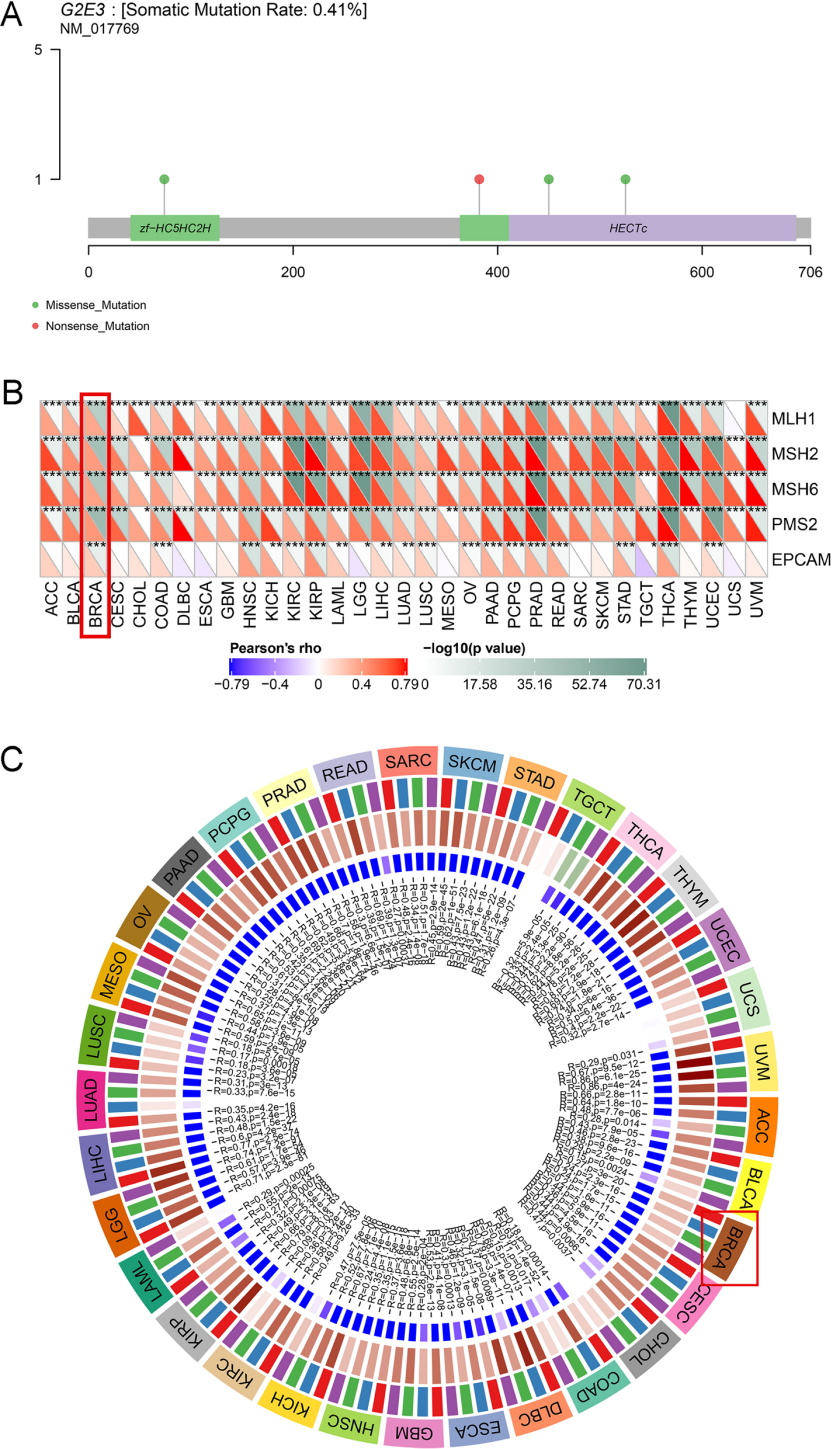
Mutations of *G2E3* gene in breast cancer. **A** The somatic mutation rate of the *G2E3* gene in breast cancer. **B** Relationship between five DNA repair genes, MLH1, MSH2, MSH6, PMS2, EPCAM mutation, and *G2E3* gene expression. **C** Correlations between *G2E3* gene expression and expression of four methyltransferases (DNMT1, red; DNMT2, blue; DNMT3A, green; DNMT3B, purple)

Using Sanger-box tools, mutations in *G2E3* in breast cancer were also explored. The somatic mutation rate of *G2E3* in breast cancer was 0.41% (Fig. 6A).

DNA repair genes are mismatched repair genes in cells. The loss of function of critical genes in this mechanism would cause irreparable DNA replication errors, leading to increased somatic mutations [36]. Sanger-box tools used the expression profile data to evaluate the correlation between five DNA repair genes: *MLH1*, *MSH2*, *MSH6*, *PMS2*, *EPCAM* mutation, and *G2E3* expression. The G2E3 expression was significantly positively related to mutations of these five genes in breast cancer (Fig. 6B).

DNA methylation can cause changes in the chromatin structure, DNA conformation, and other factors, thereby controlling gene expression. DNA methylation can only be achieved using DNA methyltransferases [37]. Therefore, we analyzed the relationship between *G2E3* expression and the expression of the four methyltransferases. In breast cancer, the *G2E3* expression was significantly positively related to the expression of these four methyltransferases (Fig. 6C).

### Screening potential therapeutic compounds which can decrease G2E3 mRNA expression for breast cancer

**Fig. 7:**
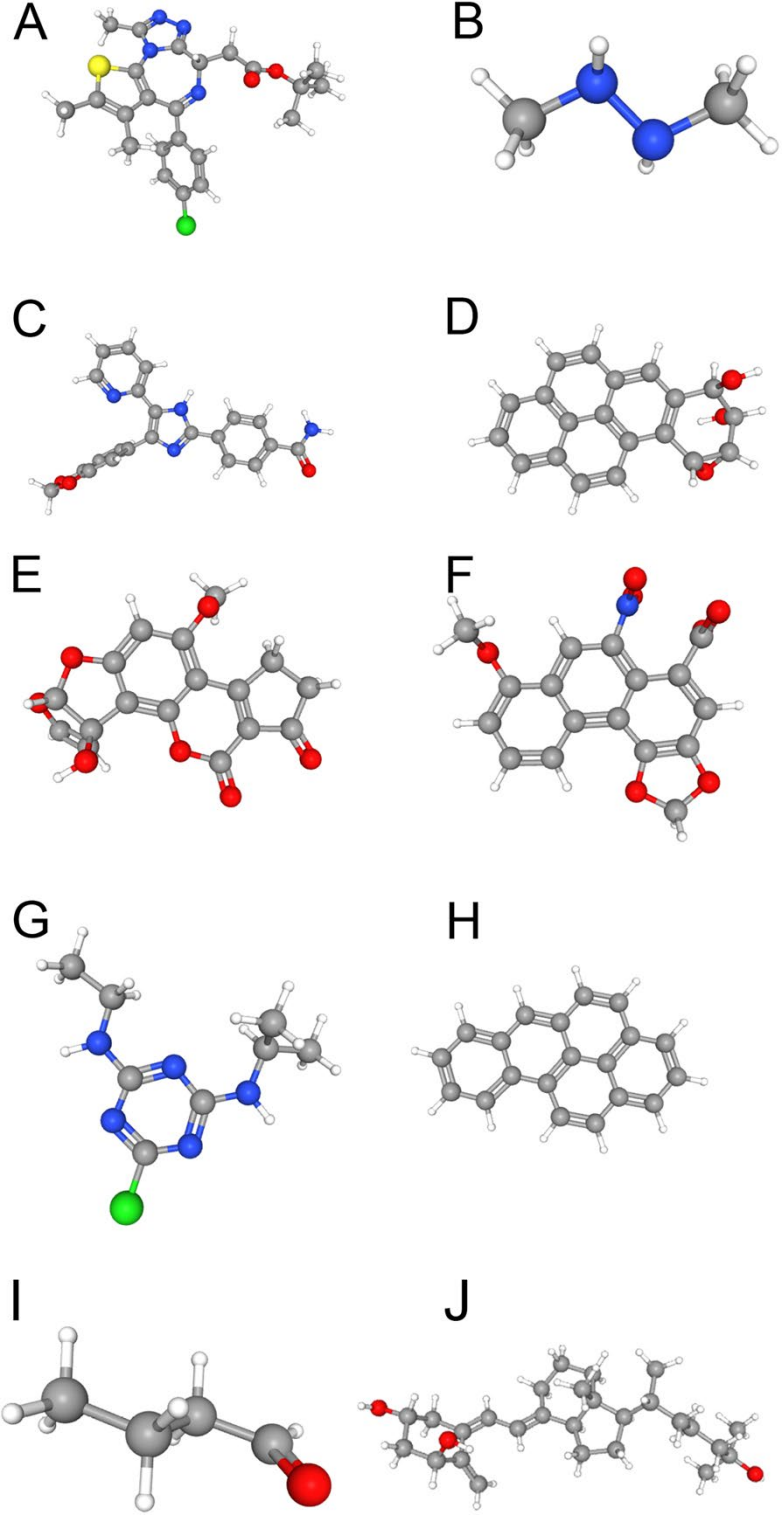
3D structures of the top ten compounds which can decrease mRNA expression of G2E3 for breast cancer gained from PubChem database

By applying the CTD database, we aimed to screen for potential therapeutic compounds that could decrease the mRNA expression of the *G2E3* oncogene in breast cancer patients. We found that (+)-JQ1 compound, 1,2-dimethylhydrazine, and other compounds can decrease the *G2E3* mRNA expression, which may be potential therapeutic compounds for breast cancer. The results are presented in Table 6. The 3D structures of the top ten compounds were obtained from the PubChem database (https://pubchem.ncbi.nlm.nih.gov) and are shown in Fig. 7.

**Table 6:** Potential therapeutic compounds that can result in decreased expression of G2E3 mRNA

Chemical name	Chemical ID	Interaction actions	Reference count	Organism count
(+)-JQ1 compound	C561695	Decreases^expression	1	1
1,2-Dimethylhydrazine	D019813	Decreases^expression	1	1
4-(5-Benzo (1, 3) dioxol-5-yl-4-pyridin-2-yl-1H-imidazol-2-yl) benzamide	C459179	Affects^cotreatment|decreases^expression	1	1
7,8-Dihydro-7,8-dihydroxybenzo(a)pyrene 9,10-oxide	D015123	Decreases^expression	2	1
Aflatoxin M1	D016607	Decreases^expression	1	1
Aristolochic acid I	C000228	Decreases^expression	1	1
Atrazine	D001280	Decreases^expression	1	1
Benzo(a)pyrene	D001564	Decreases^expression	2	1
Butyraldehyde	C018475	Decreases^expression	1	1
Calcitriol	D002117	Affects^cotreatment|decreases^expression	1	1
Cisplatin	D002945	Decreases^expression	3	2
Dicrotophos	C000944	Decreases^expression	1	1
Dietary fats	D004041	Decreases^expression	1	1
Dorsomorphin	C516138	Affects^cotreatment|decreases^expression	1	1
Doxorubicin	D004317	Decreases^expression	1	1
Epigallocatechin gallate	C045651	Affects^cotreatment|decreases^expression	1	1
Fipronil	C082360	Decreases^expression	1	1
Formaldehyde	D005557	Decreases^expression	1	1
Hexabromocyclododecane	C089796	Decreases^expression	1	1
Irinotecan	D000077146	Decreases^expression	1	1
Jinfukang	C544151	Decreases^expression	1	1
Magnetite nanoparticles	D058185	Affects^binding|decreases^expression	1	1
Methyl methanesulfonate	D008741	Decreases^expression	1	1
Methylmercuric chloride	C004925	Decreases^expression	1	1
N-(2-(1,1′-bicyclopropyl)-2-ylphenyl)-3-(difluoromethyl)-1-methyl-1H-pyrazole-4-carboxamide	C583365	Decreases^expression	1	1
Oxaliplatin	D000077150	Decreases^expression	1	1
Perfluorooctane sulfonic acid	C076994	Decreases^expression	1	1
Phenobarbital	D010634	Decreases^expression	1	1
Phenylmercuric acetate	D010662	Affects^cotreatment|decreases^expression	1	1
Potassium chromate (VI)	C027373	Decreases^expression	1	1
Quercetin	D011794	Decreases^expression	1	1
Succimer	D004113	Affects^binding|decreases^expression	1	1
Sunitinib	D000077210	Decreases^expression	1	1
Testosterone	D013739	Decreases^expression	1	1
Tetrachlorodibenzodioxin	D013749	Decreases^expression	1	1
Topotecan	D019772	Decreases^expression	1	1
Tretinoin	D014212	Decreases^expression	1	1
Trichostatin A	C012589	Affects^cotreatment|decreases^expression	1	1
Valproic acid	D014635	Decreases^expression	2	1
Vitamin K3	D024483	Decreases^expression	1	1
Vorinostat	D000077337	Decreases^expression	1	1

## Discussion

Brooks et al. first identified the G2E3 protein with a HECT (homologous to E6-associated protein) domain located on the C-terminus, suggesting that it might serve as a ubiquitin ligase or E3 [12]. They further found that G2E3 functions as a nucleocytoplasmic shuttling protein. Its export relies on sequences in the carboxy-terminal half of the protein and is not dependent on the regular CRM1/Exportin 1-mediate process. Following DNA damage, G2E3 rapidly delocalizes from the nucleoli into the cytoplasm. The cell cycle phase-specific expression and rapidly induced subcellular localization of G2E3 play an important role in regulating the cell cycle and the DDR. Owing to the rapid response to DNA damage, Schmidt et al. found that G2E3 participates in the process by which chemotherapy drugs kill tumor cells [13]. One of the main functions of chemotherapeutic drugs that kill tumor cells is DNA damage. They result in DNA double-strain breaks in cancer cells, which are often associated with cell death [38–40]. Phosphorylation of histone variant H2AX at Ser139 (γH2AX) is a marker of DDR [41]. However, in the process of cisplatin treatment and the ubiquitin system, the decrease in γH2AX is a marker of DDR. Schmidt et al. found that in cisplatin treatment, G2E3 depletion may decrease the phosphorylation of H2AX. G2E3 was required for transmitting the DDR signal to H2AX when cells were treated with cisplatin. Loss of G2E3 can cause p53 accumulation, cell apoptosis, and inhibition of tumor cell proliferation. When cells are depleted of the nucleoside analogs gemcitabine and G2E3, DNA replication can be further hindered. Simultaneously, the G2E3 expression decreased when tumor cells were treated with chemotherapeutic drugs, such as cisplatin. Therefore, they concluded that G2E3 might serve as a novel modulator of DDR, and its loss of expression can sensitize tumor cells to DNA damage. These findings predict that *G2E3* is an oncogene for breast cancer. These findings are consistent with our results.

This study found G2E3 expression was significantly higher in cancer tissues than in normal tissues, validated using an online database and PCR. By the Kaplan-Meier plotter database, we found G2E3 high expression was significantly related to shorter RFS and OS. We further validated this by breast cancer clinical specimens. We also found G2E3-positive expression had a high diagnostic value for DFS and OS by ROC curves and was an independent prognostic predictor for DFS in breast cancer patients. Pathway enrichment analysis of G2E3 revealed that it was enriched in “HALLMARK_E2F_TARGETS,” “HALLMARK_PI3K_AKT_MTOR_SIGNALING,” and other cancer-related pathways. We found that the expression of G2E3 was significantly negatively correlated with lymphocytes infiltration, which was also validated in clinical breast cancer specimens. We also explored the relationships between G2E3 expression and the tumor immune microenvironment and tumor neoantigens. Finally, we explored the mutation of the *G2E3* gene in breast cancer and screened for potential therapeutic compounds that can decrease the *G2E3* mRNA expression. We hypothesized that G2E3 could serve as a novel therapeutic target for breast cancer. However, there are still some minor issues with the results of the online database. From the results of Fig. 2 C, E, G, and H, we can find that G2E3 expression had a significant adverse effect on RFS and OS, but either stage 3 or N2 patients had the highest G2E3 expression, not stage 4 nor N3 patients which probably correlated with worse prognosis than stage 3 nor N2 patients [42–45]. We think the results may owe to the few numbers of breast cancer patients included in Fig. 2 C and E. Figure 2C only included 20-stage 4 breast cancer patients, and Fig. 2E only included 77 N3 breast cancer patients. The worse prognostic effects of *G2E3* mRNA high expression on RFS and OS presented in Fig. 2 G and H were gained based on 2032 and 943 breast cancer patients respectively. Therefore, if included more stage 4 or N3 breast cancer patients, we may get more reasonable results.

Studies have found that G2E3 is critical for early embryonic development, especially for maintaining germline stem cells in *Drosophila* [46]. Brooks et al. found that G2E3 is an unusual ubiquitin ligase essential for early embryonic [47]. Li et al. found *G2E3* methylation may affect embryo diapause by regulating the cell cycle [48]. Powell et al. found that *G2E3* knockout can also increase the bodyweight of mice [49]. This finding suggests that *G2E3* may regulate obesity. Finally, Zhang et al. found that cathepsin D can enhance invasion and metastasis in breast cancer by promoting hepsin ubiquitin-proteasome degradation. However, this regulation is mediated by cathepsin D [50]. Cathepsin D overexpression can significantly increase G2E3 expression, and blocking G2E3 expression can significantly inhibit hepsin degradation induced by cathepsin D. Immunohistochemical analysis also found G2E3 expression was significantly negatively related to the expression of hepsin and positively related to cathepsin D expression in breast cancer. This result suggests that G2E3 may regulate the malignant behavior of breast cancer cells.

This study also had some limitations. First, we did not validate the function of G2E3 using in vitro or in vivo experiments. Second, we did not explore the exact regulatory mechanisms of G2E3 in breast cancer development. Finally, we did not determine a relationship between G2E3 expression and breast cancer immunity. These limitations are the future study directions for our experiments.

## Conclusions

In conclusion, we found that G2E3 was more highly expressed in breast cancer tissues than in normal breast tissues. G2E3-positive expression was related to a worse prognosis in patients with breast cancer. Genes co-expressed with *G2E3* may be enriched in the E2F and PI3K/AKT/mTOR signaling pathways and other cancer-related signaling pathways. The G2E3 expression was significantly negatively correlated with lymphocyte infiltration. Thus, G2E3 may serve as a novel prognostic biomarker and therapeutic target for breast cancer.

## Supplementary Information

**Additional file 1: Supplementary table 1.** The basic clinicopathological characteristics of these 30 patients.

**Additional file 2: Supplementary figure 1.** The top 10 pathways gained from GSEA analysis.

**Additional file 3: Supplementary figure 2.** Relationships between G2E3 and immunity in breast cancer. A: Relationships between G2E3 expression and immunoinhibitors. B: Top 4 immunoinhibitors showing the highest correlations with G2E3 expression. C: Relationships between G2E3 expression and immunostimulators. D: Top 4 immunostimulators showing the highest correlations with G2E3 expression. E: Relationships between G2E3 expression and MHC molecules. F: Top 4 MHC molecules showing the highest correlations with G2E3 expression.

**Additional file 4: Supplementary figure 3.** Relationships between G2E3 and immunity in breast cancer. A: Relationships between chemokine and G2E3 expression. B: Top 4 chemokines showing the highest correlations with G2E3 expression. C: Relationships between chemokine receptors and G2E3 expression. D: Top 4 chemokine receptors showing the highest correlations with G2E3 expression. E: G2E3 expression in different molecular subtypes of breast cancer. F: G2E3 expression in different immune subtypes of breast cancer.

**Additional file 5: Supplementary figure 4.** Relationship between G2E3 expression and immune score such as ImmuneScore in 33 tumors.

**Additional file 6: Supplementary figure 5.** Relationship between G2E3 expression and matrix score such as StromalScore in 33 tumors.

**Additional file 7: Supplementary figure 6.** Relationship between G2E3 expression and ESTIMATE immune score such as ESTIMATEscore in 33 tumors.

**Additional file 8: Supplementary figure 7.** Relationship between G2E3 gene expression and the number of antigens in 19 tumors.

## Data Availability

These data and materials can be available from corresponding authors for rational reasons.

## Abbreviations

G2E3: G2-specific E3-like
ROC: Receiver operating characteristic
DDR: DNA damage response
GTEx: Genotype-tissue expression
DFS: Disease-free survival
OS: Overall survival
PPI: Protein-protein interactions
GSEA: Gene set enrichment analysis
TMB: Tumor mutational burden
MSI: Microsatellite instability
CTD: Comparative Toxicogenomics Database
HRs: Hazard ratios
CIs: Confidence intervals
TILs: Tumor-infiltrating lymphocytes
TCGA: The Cancer Genome Atlas
BP: Biological process
CC: Cellular component
MF: Molecular function
MHC: Major histocompatibility complex
HLA: Human leukocyte antigen
TAPBP: TAP-binding protein
