# Supplementary material for: Comprehensive analysis of the expression, prognostic significance, and regulation pathway of G2E3 in breast cancer
Source: World J Surg Oncol. 2022 Dec 15;20:398. doi: 10.1186/s12957-022-02871-0 (PMC9753372; doi:10.1186/s12957-022-02871-0)
Supplement: Supplementary file 1 — Additional file 1: Supplementary table 1. The basic clinicopathological characteristics of these 30 patients. [file 12957_2022_2871_MOESM1_ESM.docx]

Supplementary table 1 The basic clinicopathological characteristics of these 30 patients

| Clinicopathological characteristics | Patient 1 | Patient 2 | Patient 3 | Patient 4 | Patient 5 | Patient 6 |
| --- | --- | --- | --- | --- | --- | --- |
| Gender | Female | Female | Female | Female | Female | Female |
| Age | 51 | 54 | 56 | 52 | 52 | 72 |
| T grade | 3 | 3 | 2 | 3 | 2 | 1 |
| N grade | 0 | 1 | 2 | 0 | 2 | 0 |
| Menopause status | Premenopausal | Postmenopausal | Premenopausal | Postmenopausal | Postmenopausal | Postmenopausal |
| Pathological type | Invasive ductal carcinoma | Invasive ductal carcinoma | Invasive ductal carcinoma | Invasive ductal carcinoma | Invasive ductal carcinoma | Invasive ductal carcinoma |
| Subclasses | Luminal | Luminal | Luminal | HER2 positive | Triple negative | Luminal |
| Metastasis | No | No | No | No | No | No |
|  | Patient 7 | Patient 8 | Patient 9 | Patient 10 | Patient 11 | Patient 12 |
| Gender | Female | Female | Female | Female | Female | Female |
| Age | 43 | 44 | 49 | 49 | 60 | 42 |
| T grade | 2 | 1 | 1 | 1 | 1 | 2 |
| N grade | 1 | 0 | 0 | 0 | 0 | 0 |
| Menopause status | Premenopausal | Premenopausal | Premenopausal | Postmenopausal | Postmenopausal | Premenopausal |
| Pathological type | Invasive ductal carcinoma | Invasive ductal carcinoma | Invasive ductal carcinoma | Invasive ductal carcinoma | Invasive ductal carcinoma | Invasive ductal carcinoma |
| Subclasses | HER2 positive | HER2 positive | HER2 positive | HER2 positive | HER2 positive | HER2 positive |
| Metastasis | No | No | No | No | No | No |
|  | Patient 13 | Patient 14 | Patient 15 | Patient 16 | Patient 17 | Patient 18 |
| Gender | Female | Female | Female | Female | Female | Female |
| Age | 59 | 74 | 49 | 65 | 51 | 44 |
| T grade | 2 | 1 | 1 | 2 | 2 | 1 |
| N grade | 1 | 1 | 0 | 0 | 1 | 0 |
| Menopause status | Postmenopausal | Postmenopausal | Premenopausal | Postmenopausal | Premenopausal | Premenopausal |
| Pathological type | Invasive ductal carcinoma | Invasive ductal carcinoma | Invasive ductal carcinoma | Invasive ductal carcinoma | Invasive ductal carcinoma | Invasive ductal carcinoma |
| Subclasses | Luminal | Luminal | Luminal | Luminal | Luminal | Luminal |
| Metastasis | No | No | No | No | No | No |
|  | Patient 19 | Patient 20 | Patient 21 | Patient 22 | Patient 23 | Patient 24 |
| Gender | Female | Female | Female | Female | Female | Female |
| Age | 68 | 47 | 50 | 56 | 58 | 62 |
| T grade | 1 | 2 | 2 | 2 | 2 | 2 |
| N grade | 0 | 0 | 0 | 0 | 0 | 0 |
| Menopause status | Premenopausal | Premenopausal | Premenopausal | Postmenopausal | Postmenopausal | Postmenopausal |
| Pathological type | Invasive ductal carcinoma | Invasive ductal carcinoma | Invasive ductal carcinoma | Invasive ductal carcinoma | Invasive ductal carcinoma | Invasive ductal carcinoma |
| Subclasses | Luminal | Luminal | Luminal | Luminal | Luminal | Luminal |
| Metastasis | No | No | No | No | No | No |
|  | Patient 25 | Patient 26 | Patient 27 | Patient 28 | Patient 29 | Patient 30 |
| Gender | Female | Female | Female | Female | Female | Female |
| Age | 45 | 56 | 59 | 74 | 45 | 47 |
| T grade | 1 | 1 | 1 | 1 | 2 | 2 |
| N grade | 0 | 0 | 0 | 0 | 0 | 0 |
| Menopause status | Premenopausal | Premenopausal | Postmenopausal | Postmenopausal | Premenopausal | Premenopausal |
| Pathological type | Invasive ductal carcinoma | Invasive ductal carcinoma | Invasive ductal carcinoma | Invasive ductal carcinoma | Invasive ductal carcinoma | Invasive ductal carcinoma |
| Subclasses | Triple negative | Triple negative | Triple negative | Triple negative | Triple negative | Triple negative |
| Metastasis | No | No | No | No | No | No |
