# Supplementary figures and images for: Comprehensive analysis of the expression, prognostic significance, and regulation pathway of G2E3 in breast cancer
Source: World J Surg Oncol. 2022 Dec 15;20:398. doi: 10.1186/s12957-022-02871-0 (PMC9753372; doi:10.1186/s12957-022-02871-0)

Supp Fig 1

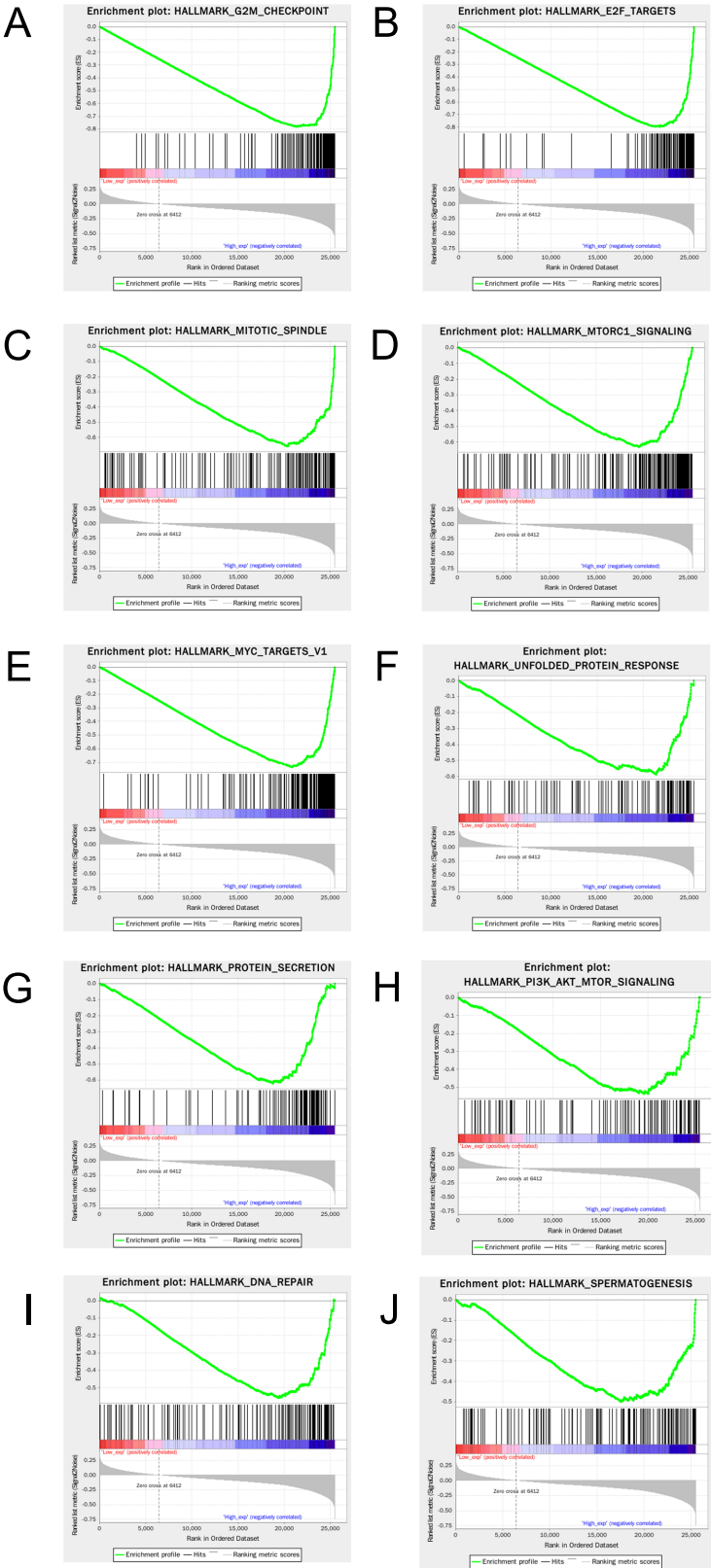

Supplement: Supplementary file 2 — Additional file 2: Supplementary figure 1. The top 10 pathways gained from GSEA analysis. [file 12957_2022_2871_MOESM2_ESM.pdf]

Supp Fig 3

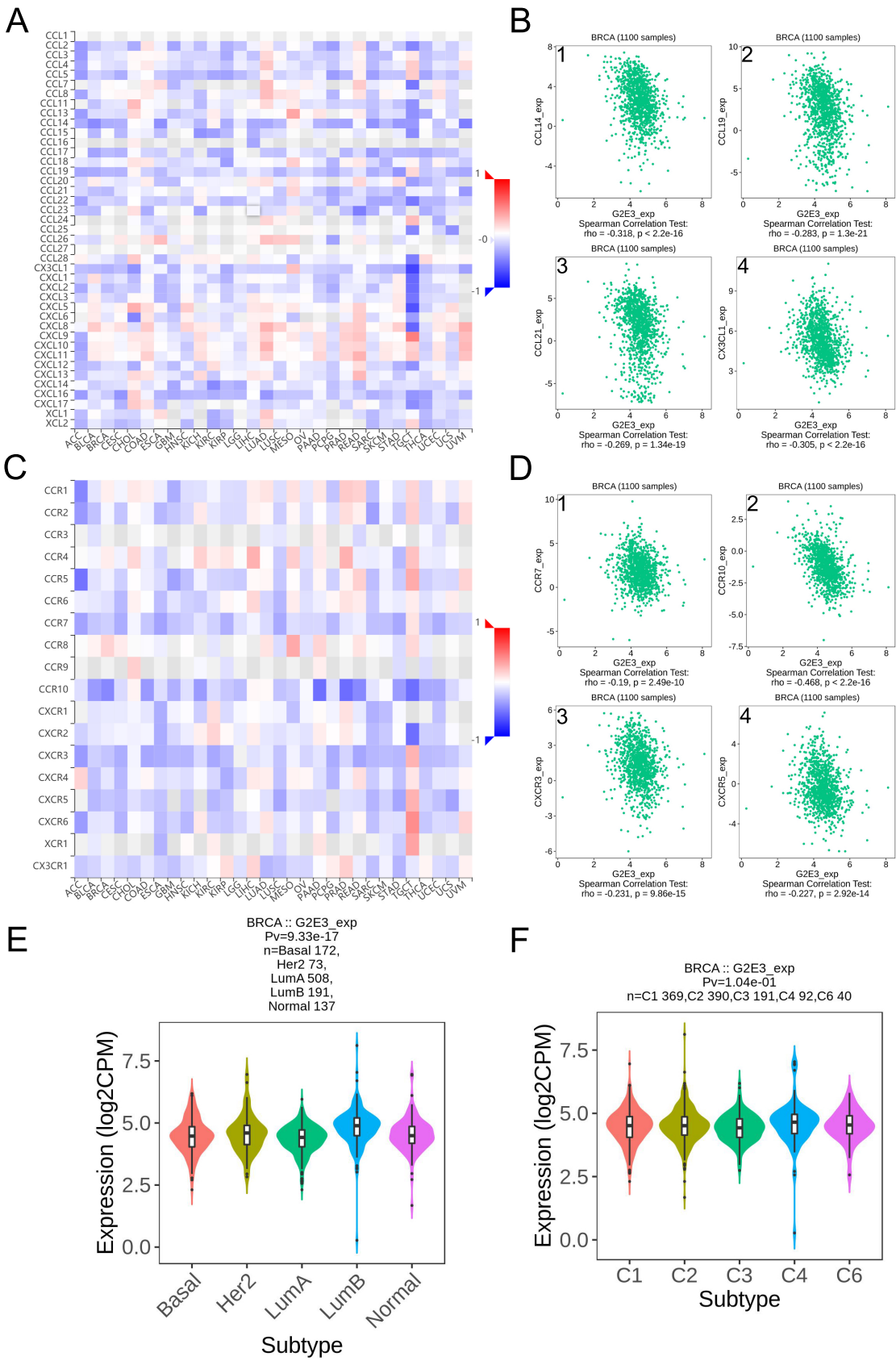

Supplement: Supplementary file 4 — Additional file 4: Supplementary figure 3. Relationships between G2E3 and immunity in breast cancer. A: Relationships between chemokine and G2E3 expression. B: Top 4 chemokines showing the highest correlations with G2E3 expression. C: Relationships between chemokine receptors and G2E3 expression. D: Top 4 chemokine receptors showing the highest correlations with G2E3 expression. E: G2E3 expression in different molecular subtypes of breast cancer. F: G2E3 expression in different immune subtypes of breast cancer. [file 12957_2022_2871_MOESM4_ESM.pdf]

## Supp Fig 4

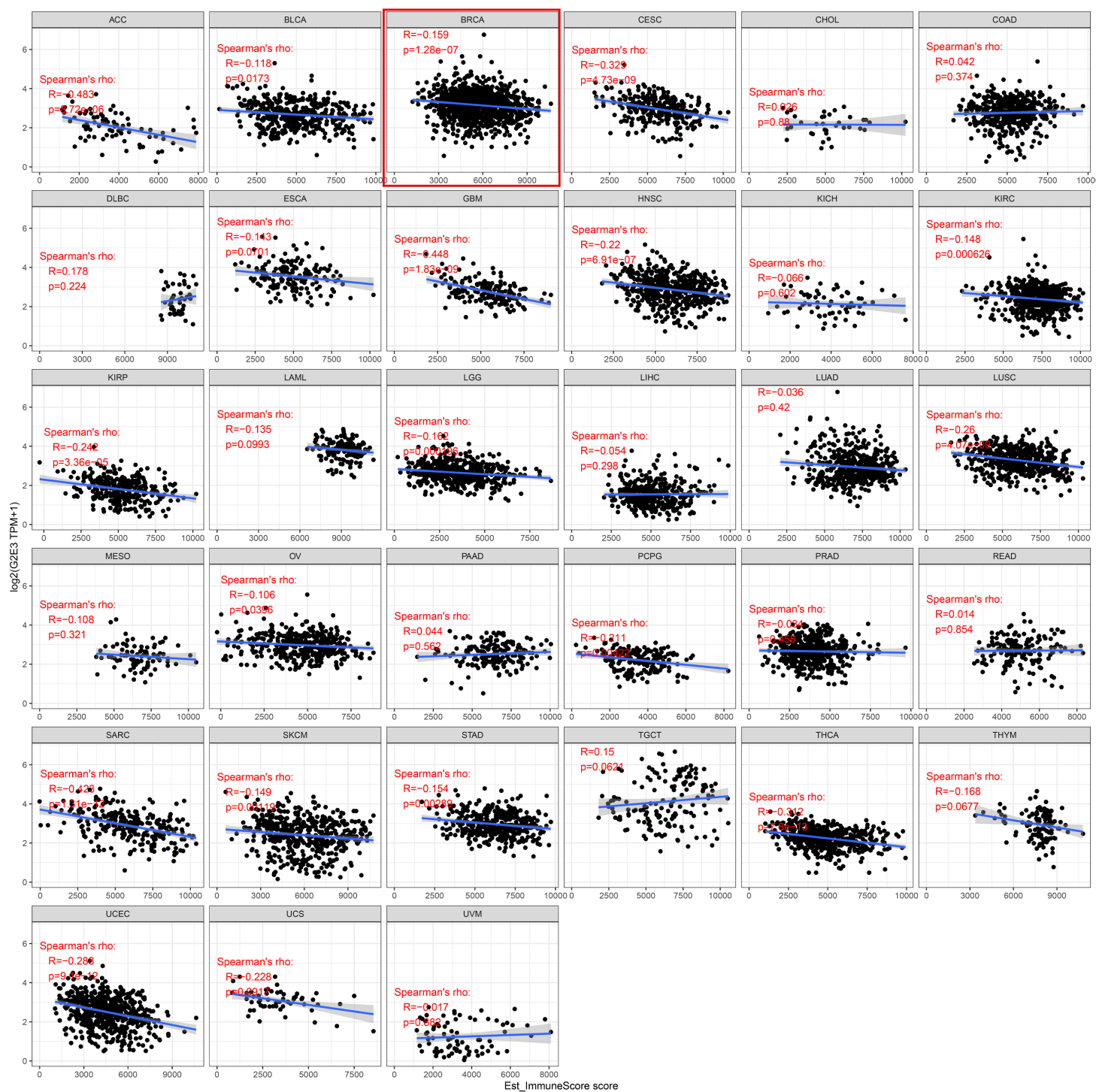

Supplement: Supplementary file 5 — Additional file 5: Supplementary figure 4. Relationship between G2E3 expression and immune score such as ImmuneScore in 33 tumors. [file 12957_2022_2871_MOESM5_ESM.pdf]

Supp Fig 5

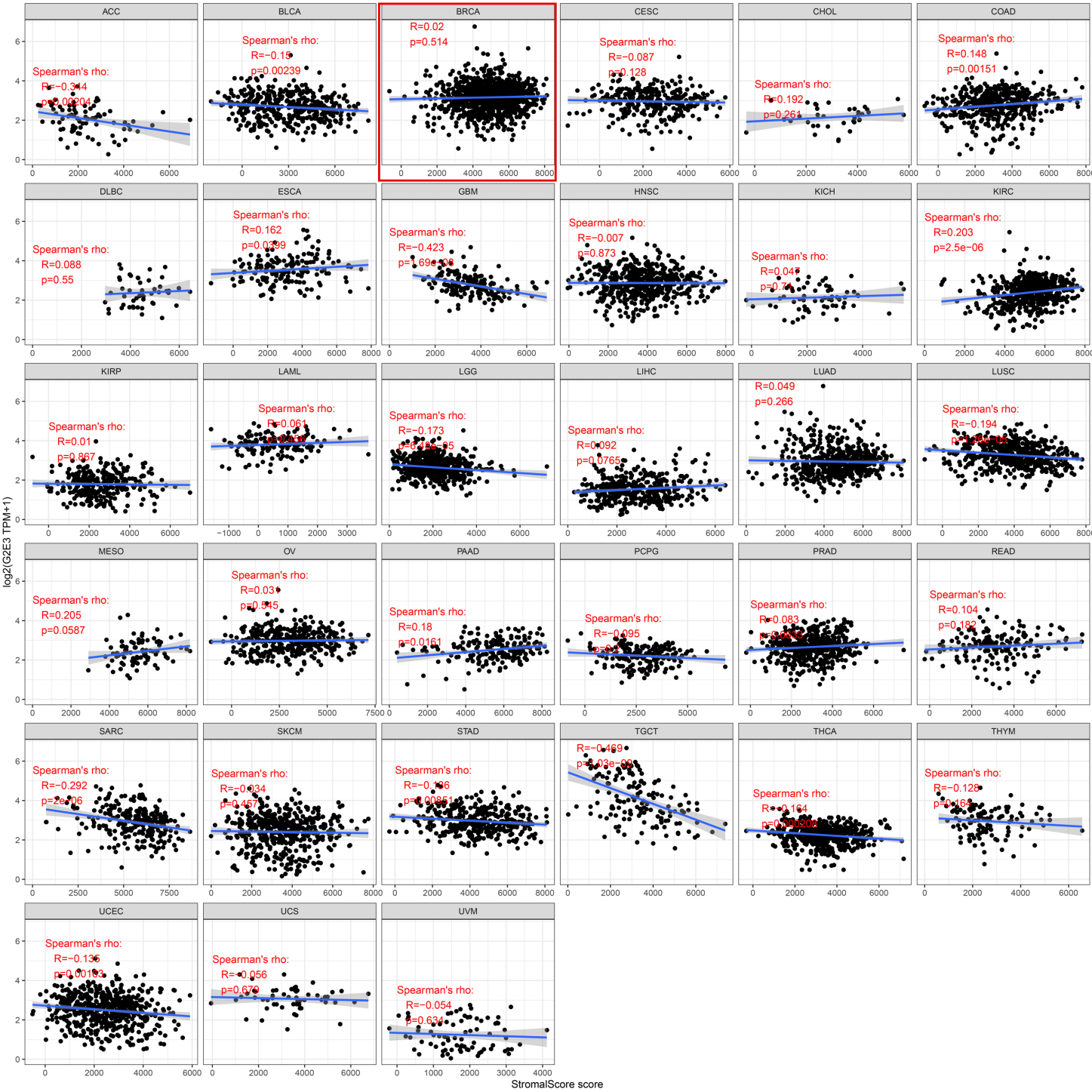

Supplement: Supplementary file 6 — Additional file 6: Supplementary figure 5. Relationship between G2E3 expression and matrix score such as StromalScore in 33 tumors. [file 12957_2022_2871_MOESM6_ESM.pdf]

Supp Fig 6

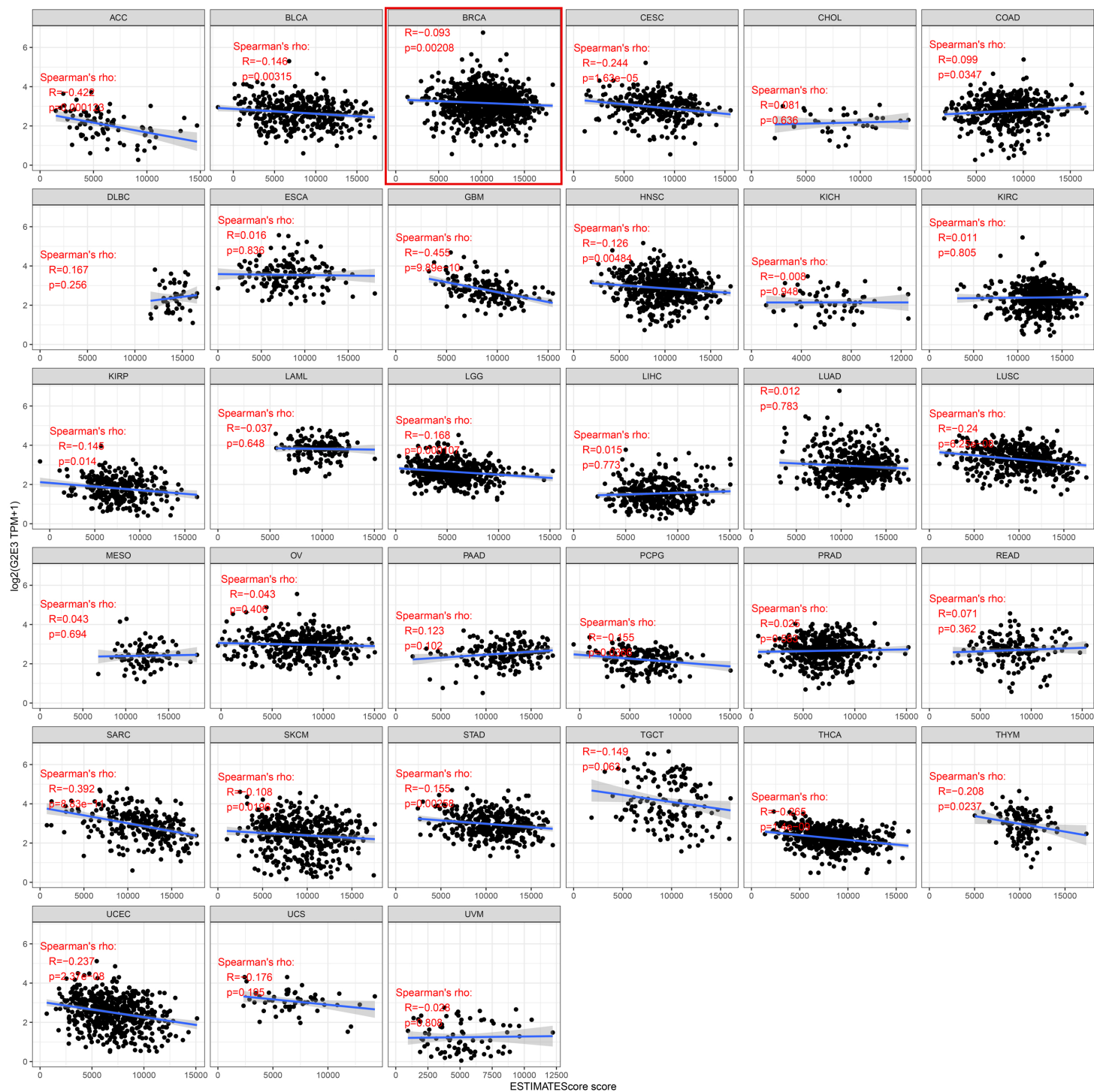

Supplement: Supplementary file 7 — Additional file 7: Supplementary figure 6. Relationship between G2E3 expression and ESTIMATE immune score such as ESTIMATEscore in 33 tumors. [file 12957_2022_2871_MOESM7_ESM.pdf]

Supp Fig 7

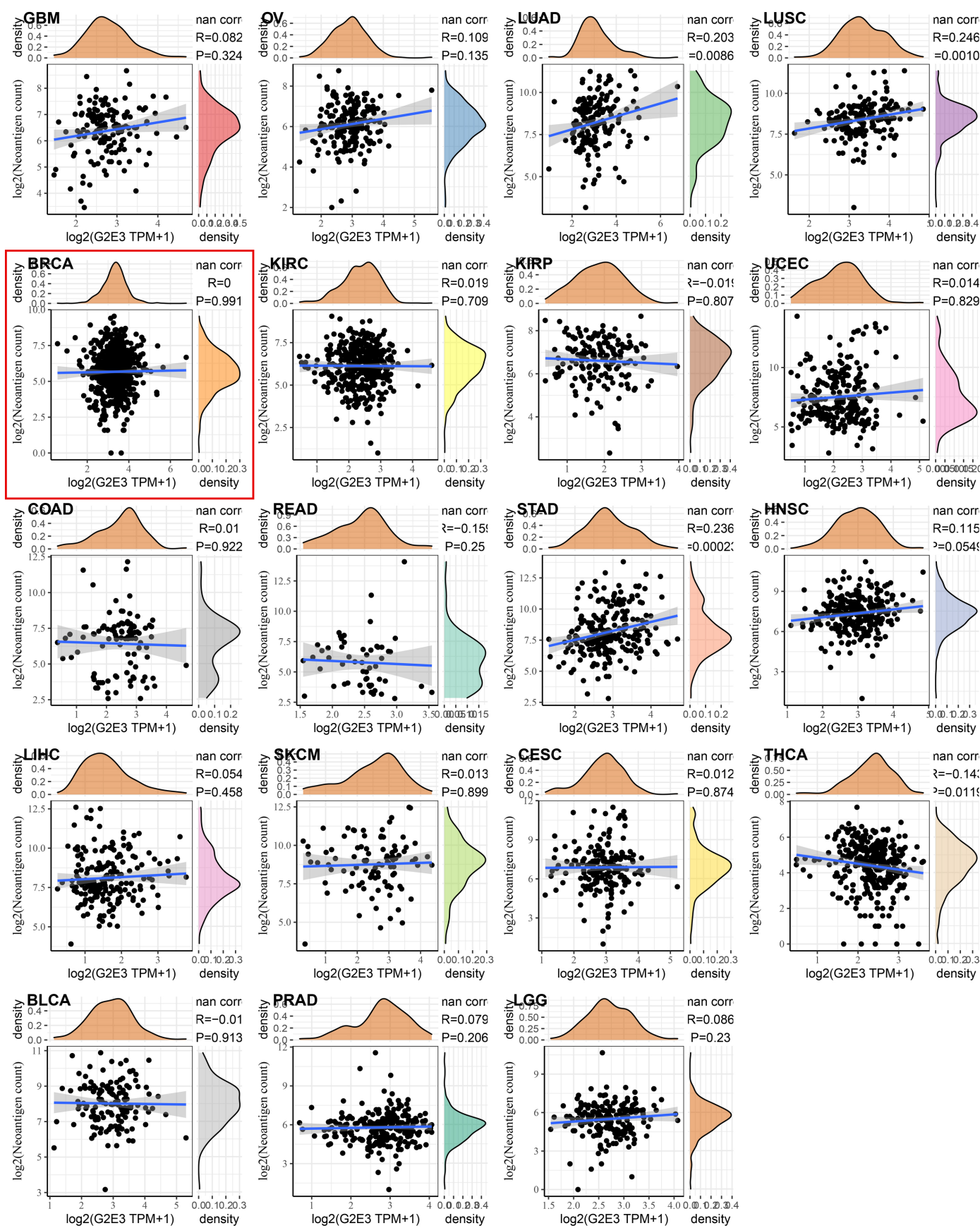

Supplement: Supplementary file 8 — Additional file 8: Supplementary figure 7. Relationship between G2E3 gene expression and the number of antigens in 19 tumors. [file 12957_2022_2871_MOESM8_ESM.pdf]
