# Supplementary material for: Comprehensive analysis of the expression, prognostic significance, and regulation pathway of G2E3 in breast cancer
Source: World J Surg Oncol. 2022 Dec 15;20:398. doi: 10.1186/s12957-022-02871-0 (PMC9753372; doi:10.1186/s12957-022-02871-0)
Supplement: Supplementary file 3 — Additional file 3: Supplementary figure 2. Relationships between G2E3 and immunity in breast cancer. A: Relationships between G2E3 expression and immunoinhibitors. B: Top 4 immunoinhibitors showing the highest correlations with G2E3 expression. C: Relationships between G2E3 expression and immunostimulators. D: Top 4 immunostimulators showing the highest correlations with G2E3 expression. E: Relationships between G2E3 expression and MHC molecules. F: Top 4 MHC molecules showing the highest correlations with G2E3 expression. [file 12957_2022_2871_MOESM3_ESM.pdf]

Supp Fig 2

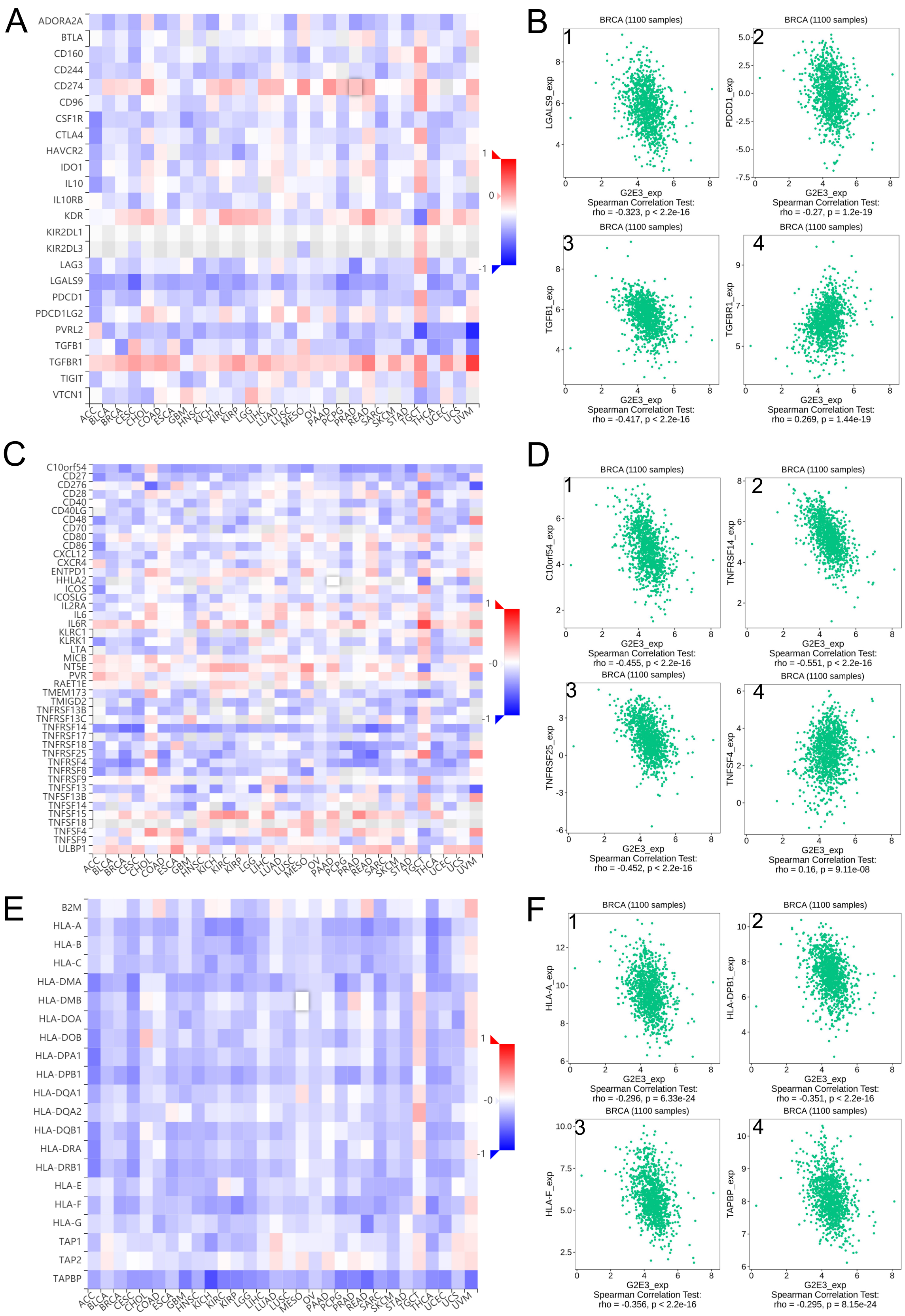
A
ADORA2A
BTLA
CD160
CD244
CD274
CD96
CSF1R
CTLA4
HAVCR2
IDO1
IL10
IL10RB
KDR
KIR2DL1
KIR2DL3
LAG3
LGALS9
PDCD1
PDCD1LG2
PVRL2
TGFB1
TGFBR1
TIGIT
VTCN1
ACC BLCA BRCA CESC CHOL COAD ESCA GBM HNSC KICH KIRC KIRP LGG LIHC LUAD LUSC MESO OV PAAD PCPG PRAD READ SARC SKCM STAD TGCT THCA UCEC UCS UVM
B
BRCA (1100 samples)
G2E3_exp
Spearman Correlation Test:
rho = -0.323, p < 2.2e-16
LGALS9_exp
rho = -0.27, p = 1.2e-19
PDCD1_exp
rho = -0.417, p < 2.2e-16
TGFB1_exp
rho = 0.269, p = 1.44e-19
TGFBR1_exp
C
C10orf54
CD27
CD276
CD28
CD40
CD40LG
CD48
CD70
CD80
CD86
CXCL12
CXCR4
ENTPD1
HHLA2
ICOS
ICOSLG
IL2RA
IL6
IL6R
KLRC1
KLRK1
LTA
MICB
NT5E
PVR
RAET1E
TMEM173
TMIGD2
TNFRSF13B
TNFRSF13C
TNFRSF14
TNFRSF17
TNFRSF18
TNFRSF25
TNFRSF4
TNFRSF8
TNFRSF9
TNFSF13
TNFSF13B
TNFSF14
TNFSF15
TNFSF18
TNFSF4
TNFSF9
ULBP1
D
C10orf54_exp
rho = -0.455, p < 2.2e-16
TNFRSF14_exp
rho = -0.551, p < 2.2e-16
TNFRSF25_exp
rho = -0.452, p < 2.2e-16
TNFSF4_exp
rho = 0.16, p = 9.11e-08
E
B2M
HLA-A
HLA-B
HLA-C
HLA-DMA
HLA-DMB
HLA-DOA
HLA-DOB
HLA-DPA1
HLA-DPB1
HLA-DQA1
HLA-DQA2
HLA-DQB1
HLA-DRA
HLA-DRB1
HLA-E
HLA-F
HLA-G
TAP1
TAP2
TAPBP
F
HLA-A_exp
rho = -0.296, p = 6.33e-24
HLA-DPB1_exp
rho = -0.351, p < 2.2e-16
HLA-F_exp
rho = -0.356, p < 2.2e-16
TAPBP_exp
rho = -0.295, p = 8.15e-24
